# Small Molecule-Based Enzyme Inhibitors in the Treatment of Primary Hyperoxalurias

**DOI:** 10.3390/jpm11020074

**Published:** 2021-01-27

**Authors:** Maria Dolores Moya-Garzon, Jose Antonio Gomez-Vidal, Alfonso Alejo-Armijo, Joaquin Altarejos, Juan Roberto Rodriguez-Madoz, Miguel Xavier Fernandes, Eduardo Salido, Sofia Salido, Monica Diaz-Gavilan

**Affiliations:** 1 Departamento de Química Farmacéutica y Orgánica, Facultad de Farmacia, Campus de Cartuja s/n, 18071 Granada, Spain; mdmoya@stanford.edu (M.D.M.-G.); jagvidal@ugr.es (J.A.G.-V.); 2 Department of Pathology, Stanford University School of Medicine, Stanford, CA 94305, USA; 3 Stanford ChEM-H, Stanford University, Stanford, CA 94305, USA; 4 Departamento de Química Inorgánica y Orgánica, Facultad de Ciencias Experimentales, Campus de Excelencia Internacional Agroalimentario (ceiA3), Universidad de Jaén, 23071 Jaén, Spain; aaa00010@red.ujaen.es (A.A.-A.); jaltare@ujaen.es (J.A.); 5 Programa de Medicina Regenerativa, CIMA Universidad de Navarra, 31008 Pamplona, Spain; jrrodriguez@unav.es; 6 Instituto de Investigación Sanitaria de Navarra, IdiSNA, 31008 Pamplona, Spain; 7 Instituto Universitario de Bio-Orgánica “Antonio González”, Instituto de Tecnologías Biomédicas, Universidad de La Laguna, 38206 La Laguna, Spain; mfernand@ull.edu.es; 8 Hospital Universitario de Canarias & Center for Rare Diseases (CIBERER), 38320 Tenerife, Spain; esalido@ull.es

**Keywords:** hyperoxaluria, oxalate, inhibitor, small molecule drug, glycolate oxidase, lactate dehydrogenase, liver selective distribution

## Abstract

Primary hyperoxalurias (PHs) are a group of inherited alterations of the hepatic glyoxylate metabolism. PHs classification based on gene mutations parallel a variety of enzymatic defects, and all involve the harmful accumulation of calcium oxalate crystals that produce systemic damage. These geographically widespread rare diseases have a deep impact in the life quality of the patients. Until recently, treatments were limited to palliative measures and kidney/liver transplants in the most severe forms. Efforts made to develop pharmacological treatments succeeded with the biotechnological agent lumasiran, a siRNA product against glycolate oxidase, which has become the first effective therapy to treat PH1. However, small molecule drugs have classically been preferred since they benefit from experience and have better pharmacological properties. The development of small molecule inhibitors designed against key enzymes of glyoxylate metabolism is on the focus of research. Enzyme inhibitors are successful and widely used in several diseases and their pharmacokinetic advantages are well known. In PHs, effective enzymatic targets have been determined and characterized for drug design and interesting inhibitory activities have been achieved both in vitro and in vivo. This review describes the most recent advances towards the development of small molecule enzyme inhibitors in the treatment of PHs, introducing the multi-target approach as a more effective and safe therapeutic option.

## 1. Introduction

### 1.1. Primary Hyperoxalurias: Pathology and Current Treatment

Primary hyperoxaluria (PH) is a rare disease of liver metabolism that results in excess oxalate production and urine excretion (hyperoxaluria). Its estimated prevalence in clinical studies is around 1–3 per million population [1], although recent genomic investigations suggest significant underdiagnosis [2]. Indeed, genomic studies identify such number of mutant alleles in the general population that a prevalence of one in 58,000 individuals could be a good estimate [2]. This severe disease is caused by genetic changes that alter glyoxylate and hydroxyproline metabolism resulting in overproduction of oxalate by the liver [3]. Other situations in which elevated oxalate in the urine is due to excessive intake or absorption of oxalate or its precursor are known as secondary hyperoxalurias.

PH1 (OMIM #259900) is the most common and severe form of PH, due to mutations in the *AGXT* gene (coding for alanine: glyoxylate amino-transferase, AGT) [4] and accounts for about 80% of PH cases. Other hereditary hyperoxalurias include PH2 (OMIM #260000), caused by mutations in the *GRHPR* gene (coding for glyoxylate/hydroxypyruvate reductase, GRHPR) and PH3 (OMIM #613616), caused by mutations in the *HOGA1* gene (coding 4-hydroxy-2-oxoglutarate aldolase 1, HOGA1). All three forms of PH are inherited as autosomal recessive diseases and the possibility exists that additional types of PH (non PH1-PH3) might be described in the future.

**Figure 1:**
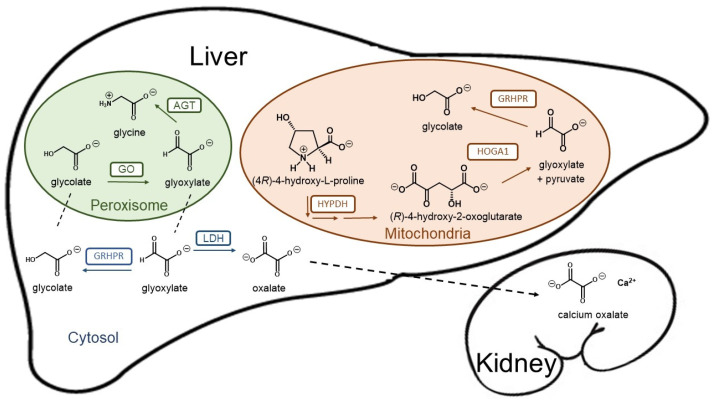
Schematic representation of the hepatic glyoxylate metabolism and calcium oxalate formation. Glyoxylate is a very reactive aldehyde produced in the intermediary metabolism of glycine, (4*R*)-4-hydroxy-L-proline and glycolate as the best known sources in humans. Lactate dehydrogenase (LDH) oxidizes cytosolic glyoxylate into oxalate, an end product of metabolism that can precipitate as tissue-damaging calcium oxalate. Two organelles play crucial roles in glyoxylate detoxification. Alanine-glyoxylate aminotransferase (AGT) plays a central role converting glyoxylate into glycine in the peroxisome, while mitochondrial and cytosolic glyoxylate can be reduced to glycolate by glycolate reductase/hydroxypyruvate reductase (GRHPR), preventing excessive oxidation to oxalate by LDH. Mitochondrial hydroxyproline metabolism via hydroxyproline dehydrogenase (HYPDH) results in the production of (*R*)-4-hydroxy-2-oxoglutarate that is normally split into glyoxylate and pyruvate by 4-hydroxy-2-oxoglutarate aldolase 1 (HOGA1).

Loss of function mutations in any of these three genes result in a deficit to detoxify glyoxylate, which is then converted into oxalate by hepatic lactate dehydrogenase (LDH). Since humans have no enzyme capable of degrading oxalate, this dicarboxylic compound must be eliminated primarily by the kidneys, where it can complex with calcium to form crystals and calcium oxalate (CaOx) stones (Figure 1). Patients with primary hyperoxaluria experience high concentrations of oxalate in the urine from birth. PH1 patients typically yield oxalate excretion > 1 mmol/1.73 m^2^ per day (normal range < 0.5 mmol) [5]. The increased urinary excretion of oxalate results in urinary CaOx supersaturation, which leads to crystal aggregation, urolithiasis, and/or nephrocalcinosis. Nephrocalcinosis or recurrent urolithiasis can cause renal tubulointerstitial inflammation and fibrosis and, if persistent, end-stage kidney disease. In addition, urolithiasis complications such as infection and obstruction and the surgical interventions to treat urolithiasis, also contribute to renal damage in affected patients.

**Figure 2:**
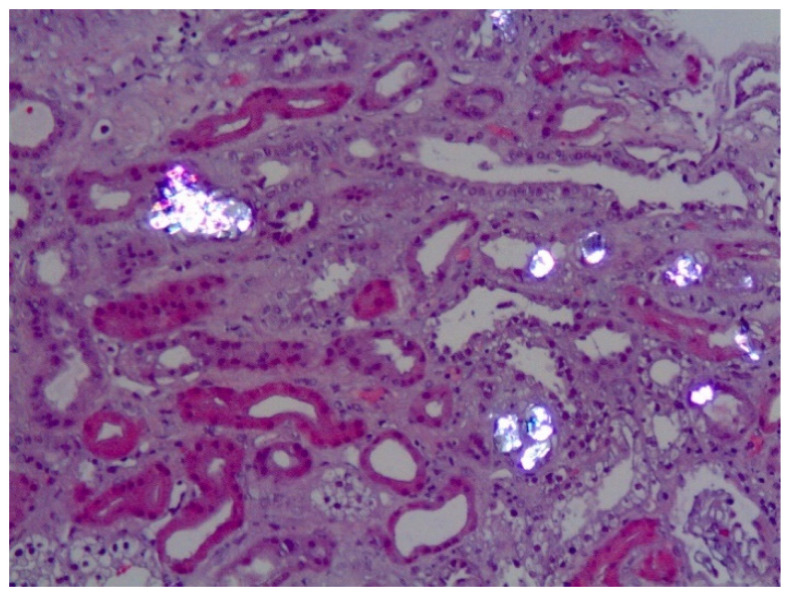
Calcium oxalate crystals (bright birefringent crystals) are shown in a kidney biopsy of a PH1 patient with chronic renal disease. Note the tubular damage and atrophy, interstitial inflammation and fibrosis secondary to calcium oxalate deposits.

The typical clinical presentation is either recurrent kidney stone episodes at a young age and/or chronic renal failure. In many instances, most frequently in PH1, oxalate nephropathy results in end stage renal disease (ESRD) (Figure 2). The consequences of declining kidney function can be particularly severe in these conditions, since the oxalate load can no longer be eliminated, and plasma oxalate levels rise to life-threatening levels [1]. In PH patients with kidney failure, dialysis cannot remove enough oxalate to keep up with daily production, resulting in oxalate deposition in skin, retina, heart, vessels, bones and other organs (systemic oxalosis), leading to severe morbidity and death. 

Current strategies to reduce kidney damage try to reduce CaOx crystal formation through high urine volume and medications such as citrate and magnesium, and in some PH patients pharmacologic doses of pyridoxine can reduce oxalate production [6]. Very large daily fluid intake is required as well as medications taken multiple times daily, compromising quality of life. Further, while these treatments may decrease the effects of hyperoxaluria, in most PH patients they do not eliminate recurring stones or ESRD. The burden of frequent symptomatic kidney stones and their associated pain, hospitalizations and need for interventional procedures, and the burden of ESRD, intensive dialysis, systemic oxalate deposition, and transplantation is enormous. Kidney transplantation alone has a high failure rate due to oxalate injury. Thus, for the severe forms of PH combined liver and kidney transplantation is the only curative treatment to date but it has significant morbi-mortality and problems associated with donor organ shortage and life-long immunosuppressive treatment [6].

Substrate reduction therapy (SRT) targeting glycolate oxidase (GO) was proposed as a novel therapeutic approach [7,8] with no nocive effects [9,10,11]. Recently, the FDA and EMA agencies have approved the first pharmacological treatment for PH1, based on siRNA inhibition of GO (lumasiran), after it has revealed promising results, meeting its primary efficacy endpoint and all tested secondary endpoints. 

### 1.2. Therapeutic Approaches in Development against Primary Hyperoxalurias: Brief Overview

The arrival of lumasiran to the market means an important achievement towards the efficient pharmacological treatment of PH1 and constitutes the third example of siRNA drug approved for clinical use [12,13].

Many other therapeutic approaches are being currently developed against PHs in general and against PH1 specifically, being this one the most severe type of PH. Such approaches include gene/protein/cell therapies, small drug administration (chaperones and enzyme inhibitors) or the use of oxalate degrading bacteria or enzymes. These strategies are aimed at different targets, whether focused on producing a decrease of the plasmatic oxalate or on minimizing the renal damage subsequent to CaOx crystallization. An interesting classification of the therapeutic approaches attending to the organs or systems at which they are targeted can be found in the recent review by Kletzmayr et al. [14]. 

**Figure 3:**
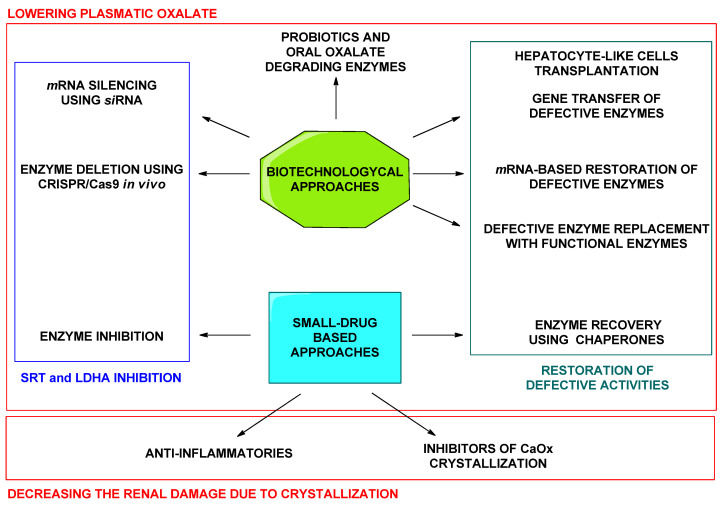
Summary of biopharmaceuticals and small drug approaches under development for the treatment of PHs.

In summary, both, biotechnological therapeutic agents and small-molecule drugs, are under exploration for the treatment of PHs (Figure 3). Small molecule drugs constitute a classical approach and, in contrast to biopharmaceuticals, present the advantage of possible oral administration and, in general, lower production costs. Besides, more is known about the possible secondary outcomes upon chronic administration or administration to children [15]. Enzymatic inhibition using small drugs is a successful approach for the treatment of uncountable diseases. Being small drugs a promising therapeutic option, in this review we want to summarize the attention that is currently being paid to small-molecule drugs in the developing treatments for PHs. 

#### 1.2.1. Therapeutic Approaches Aimed at the Lowering of the Oxalate Plasmatic Concentration

##### Recovery of Defective Activity

The aim of this approach is normalizing the glyoxylate altered metabolism by recovery of the defective activity. 

Current research on cell therapy against PHs, pursues the transplantation of genetically modified autologous hepatocyte-like cells (HLCs), which are obtained from pluripotent stem cells of PH patients (PH-iPSCs). In preliminary investigations for PH1 treatment, these PH1-iPSCs have been successfully transformed ex vivo with a lentiviral vector encoding wild-type AGT, to obtain HLCs with significant AGT expression [16]. In this sense, CRISPR/Cas9 technology has become an essential tool to deliver *Agxt* gene in PH1-iPSCs [17,18]. Gene therapy is also being investigated as a therapeutic option for PH1 at this level [19]. Major progress has been made towards in vivo delivery of AGT. Effectively, injection of liver-directed vectors encoding AGT to AGT-deficient mice decreased the urinary oxalate and prevented oxalate crystalluria [20,21,22]. Protein therapy with wild-type AGT is another therapeutic option under research for PH1. In this direction, AGT variants with enhanced stability have been obtained leading the way to enzyme-replacement therapy in PH1 [23]. Besides, polypeptide-based AGT conjugates have successfully been internalized in the cellular model of PH1 CHO-GO, restoring AGT activity inside the peroxisomal compartment [24]. Moreover, against PH1, AGT mRNA constructs have been screened in vitro and in wild-type mice for the production of a functional AGT enzyme. Up to 40% reduction in urinary oxalate has been observed using this methodology, suggesting that mRNA encoding AGT led to increased expression and activity of the AGT enzyme in liver [25]. 

The recovery of the defective AGT activity is also being addressed by the use of small drugs. In this case, pharmacological chaperones have been observed to promote the correct folding of AGT and its correct localization in the subcellular compartments [26,27,28,29,30]. Pyridoxine is currently used in the treatment of PH1 as it is able to rescue the effect of misfolding mutations of AGT, though its effectivity is only associated to certain folding-defective AGT variants [31,32]. Pyridoxamine and pyridoxal can exert the same stabilizing effect [33], and so does aminooxoacetic acid [34,35]. 

##### Substrate Reduction Therapy (SRT)

Another important strategy is the inhibition of key enzymes involved in the production of glyoxylate. The aim is decreasing the hepatic concentration of glyoxylate, which is the substrate giving rise to oxalate formation in PHs. This SRT is being developed at the level of GO [7] and hydroxyproline dehydrogenase (HYPDH) inhibition [36,37]. While the first target gives rise to useful drugs against PH1, the second one is aimed against PH2 and PH3 [38,39]. GO catalyses the formation of glyoxylate from glycolate in the hepatic peroxisomes, where AGT is in charge of detoxifying glyoxylate in physiological conditions (Figure 1). In fact, silencing of GO mRNA with siRNA is the successful mechanism of the approved drug lumasiran [40,41]. Other results support the success of this strategy against PH1 in different studies in vivo (*Agxt1^−/−^* mice) [15,42,43,44]. Another biotechnological approach in exploration for GO silencing is the GO deletion using in vivo CRISPR/Cas9 technology [45,46]. However, GO inhibition is also being assessed with small-molecule drugs [47,48,49,50,51]. Recently, furylsalicylic derivatives have been reported to decrease oxalate production in *Agxt1^−/−^* mouse hepatocytes, being this phenotypic effect to some extent related to GO inhibition. However, a multiple-target mechanism was suggested for these molecules, possibly including lactate dehydrogenase A (LDHA) inhibition [49]. Other type of recently reported GO inhibitors (GOi’s) with nanomolar activity present triazole core structure [51]. 

More enzymatic inhibitors in development for SRT are HYPDH inhibitors [52]. HYPDH is the first enzyme in the hydroxyproline catabolism in liver and kidney towards glyoxylate formation, which is detoxified by the enzyme GRHPR in mitochondria and cytosol (Figure 1). Double *Grhpr* KO (PH2 model) and *Prodh2* KO (HYPDH KO) mice showed no CaOx crystal deposition in kidneys when challenged with hydroxyproline in diet, supporting the utility of HYPDH as a target against PHs [39]. HYPDH inhibition by small molecules is under development and general structures of these molecules are available [52]. However, the structural information so far is not too specific and, for that reason, HYPDH inhibition is no further detailed in this review. 

##### Lactate Dehydrogenase A (LDHA) Inhibition

Another possibility is the prevention of oxalate formation by direct inhibition/silencing of the enzyme in charge of its formation from the accumulated glyoxylate, the hepatic isozyme LDHA (Figure 1) [53,54]. Following this strategy, useful therapeutic agents might be found against the three types of PHs. In this sense, biotechnological agents (siRNA) as well as small inhibitors are being studied as potential drugs [55,56,57]. 

##### Regulating Oxalate Uptake/Secretion at Intestinal Level

Oral administration of probiotic bacteria or probiotics-derived factors are alternative therapeutic approaches under development against PHs [58,59,60,61,62]. These agents can degrade dietary oxalate, preventing its absorption at intestinal level. The consequence is a decrease of plasmatic and thus, urinary oxalate. Some species of *Lactobacillus* and *Bifidobacterium* are being studied [60,63,64]. Especially interesting is *Oxalobacter formigenes* as, besides its oxalate-degrading activity, it has been proved to be effective stimulating oxalate secretion by the intestinal epithelium helping its clearance from plasma, in PH1 mice [61,65,66] and human PH patients [67,68,69]. 

#### 1.2.2. Therapeutic Approaches Aimed at the Minimization of the Renal Damage Provoked by CaOx Crystallization

The loss of renal functionality in PHs, is related to the inflammatory response produced by CaOx crystals deposition in the renal tubular cells, as it leads to chronic fibrogenesis. Thus, the anti-inflammatory therapy has been suggested as a therapeutic strategy in PHs as well as in other crystal accumulation pathologies [70,71]. It has been demonstrated that the inhibition of NLRP3, a component of the NALP3-inflammasome, using small molecules reduces the production of pro-inflammatory cytokines IL-1*β* and IL-18 and this effectively prevents kidney fibrosis and attenuates renal inflammation in mouse models, without immunosuppressive side effects [72]. The same way, small antagonists of IL-1*β* [73] and TNF-α [74] receptors, have been seen to protect from CaOx nephropathy in mice.

Inhibition of CaOx crystallization is another strategy under research aimed at the elimination of the renal damage caused by crystal deposition [75,76,77]. In this sense, important advances have been made recently, with the discovery of a new powerful class of CaOx inhibitors based in multivalent inositol phosphate molecules that can inhibit the crystallization process as well as the CaOx-cell interactions that lead to kidney damage [78]. 

## 2. Enzyme Inhibitors for the Treatment of PHs

### 2.1. Glycolate Oxidase Inhibitors

Glycolate oxidase (GO; EC 1.1.3.15) is a FMN-dependent flavoenzyme that belongs to the α-hydroxy acid oxidase family [79,80], which also includes *Pseudomonas putida* mandelate dehydrogenase (MDH), the flavin-binding domain of yeast flavocytochrome b_2_ (FCB2), rat long chain hydroxy acid oxidase (LCHAO), and spinach glycolate oxidase (sGO) [81]. GO is present both in mammals and plants and is localized in the peroxisome [82]. 

In 1953, GO was for the first time identified as the enzyme responsible for the oxidation of glycolic and l-lactic acids in plants [83]. The first structure of GO was elucidated from spinach [84,85] and the application of GO inhibition as herbicide treatment led to an increased interest in the discovery of GOi’s [85]. GO is a key enzyme in photorespiration, one of the main carbon metabolism pathways in plants that competes with photosynthetic CO_2_ fixation, therefore limiting CO_2_ uptake. Within this pathway, ribulose-1,5-bisphosphate carboxylase/oxygenase (Rubisco) uses O_2_ instead of CO_2_ and produces 2-phosphoglycolate, a toxic metabolite which is detoxified thanks to the glycolate-glyoxylate metabolism, in which GO takes part in the oxidation of glycolate to glyoxylate [86,87]. Thus, GO inhibition results in an increase of CO_2_ uptake from photosynthesis [88].

In higher organisms, GO was initially described in rat [89,90] and in pig livers [91,92]. Later, human glycolate oxidase (hGO) was purified and characterized [82,93] and its structure became available [81]. hGO is encoded by *HAOX1* gene (chromosome 20p12.3) and is a homotetramer composed by four 41 KDa monomers, each of them containing 370 amino acids [82,94]. In humans, GO is mainly expressed in liver and catalyzes the FMN-dependent oxidation of glycolate to glyoxylate and from glyoxylate to oxalate, contributing to stone formation in PHs [82,95].

GO has a low substrate specificity, which confers this enzyme the ability to oxidize small molecules such as glycolate and glyoxylate, but also long chain α-hydroxy acids including 2-hydroxy octanoate and 2-hydroxy palmitate [81]. The general reaction catalyzed by GO can be divided into two half-reactions. In the first half-reaction, glycolate is oxidized to glyoxylate and the flavin is reduced. In the second half reaction, the cofactor FMN is reoxidized by the action of O_2_, resulting in the formation of H_2_O_2_ [85,96].

**Figure 4:**
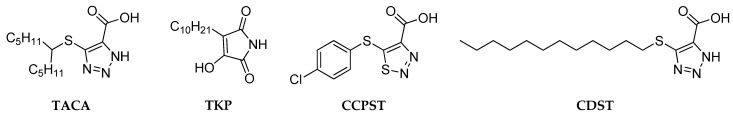
Structure of the known GOi’s TACA, TKP, CCPST and CDST.

GOi’s were traditionally developed to exploit their herbicide action, which led to the structural study of sGO [84,85]. This allowed subsequent crystallization studies of sGO with the GOi’s 4-(1-pentylhexylthio)-1*H*-1,2,3-triazole-5-carboxylic acid (TACA) and 3-decyl-2,5-dioxo-4-hydroxy-3-pyrroline (TKP), which were developed as agrochemicals (Figure 4) [85]. Once the crystal structure of hGO was available [81] crystallization studies were conducted with the reference GOi’s 4-(dodecylthio)-1*H*-1,2,3-triazole-5-carboxylic acid (CDST) [81] and 5-(4-chlorophenylthio)-1,2,3-thiadiazole-4-carboxylic acid (CCPST) [97] (Figure 4). 

Two binding regions can be distinguished for GOi’s. According to crystallographic data [81,97], the carboxyl groups of CDST and CCPST mimic glyoxylate interactions at the active site of hGO, interacting with Arg167, Arg263, and Tyr26. Their N3 atoms located at the heterocyclic rings interact with His260 overlaying with the keto group of glyoxylate. However, given that the thiadiazol ring of CCPST does not carry a proton, the interaction of this molecule would require protonation of His260, and this would contribute to the weaker binding of CCPST compared to CDST [98]. Additionally, hydrogen bonds to Tyr132 are also established through the N2 atoms of CCPST and CDST [98]. This group of amino acids constitutes the first of the two binding regions for GOi’s. Conversely, hydrophobic interactions are established through the side chain of the inhibitors with the second binding region. In the case of CCPST and CDST, these types of interactions are established with the residues Trp110, Leu205, and Tyr208, but the amino acids of the second binding region vary for each GOi [81,97]. Moreover, the flexibility of the side chains, like in the case of CDST, seems to provide a better accommodation of the inhibitor [98]. Altogether, these crystallographic studies revealed the importance of the conserved amino acids Tyr26, Trp110, Tyr132, Arg167, Lys236, His260, and Arg263 at the active site of GO and some structure-activity relationships could be established [81,99,100,101].

**Figure 5:**
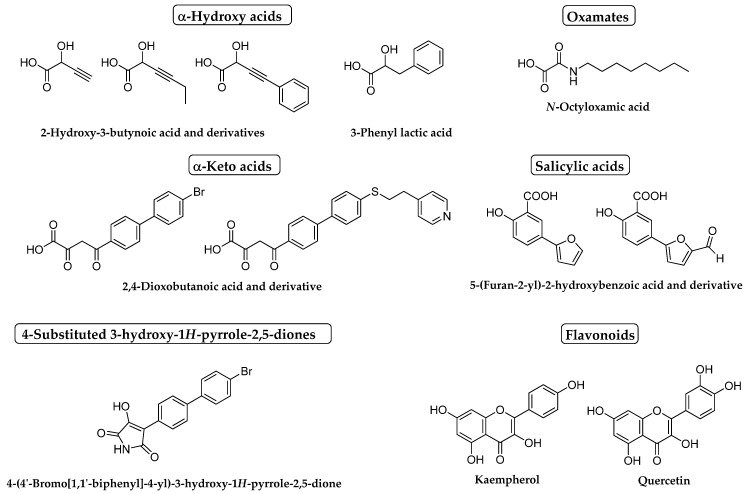
Structure of some known GOi’s belonging to different structural families.

Hence, established GOi’s share structural features including a polar head and a side chain. The polar head, which bears an acidic functionality, enters the active site and mimics the substrate interactions with the cited key amino acids, whilst the side chain, whether aromatic or aliphatic, hangs from the polar head and remains in the access channel of the enzyme establishing hydrophobic interactions and causing a disorder that prevents the adoption of the closed state. Besides, the stabilization of the ternary complex enzyme-cofactor-substrate is enhanced by the presence of flat, electron rich fragments in the polar head that establish π-π interactions with FMN flavin ring [81]. In the polar head, the presence of a protonated heteroatom located in β with respect to the acidic function has shown to be beneficial [97,98]. Different polar heads have already been explored so far, including α-hydroxy acids [93,99,102], α-keto acids [100], oxamates [93,99] and salicylic acids [49] (Figure 5). 

#### Glycolate Oxidase Inhibitors in the Treatment of PHs

Once the role of GO in glyoxylate metabolism was suggested [103], the possibility to use GOi’s for the treatment of PH1 was contemplated and multiple small-molecules acting as GOi’s have been studied so far. However, attempts at finding an effective treatment for PH1 based on small-molecule GOi’s have failed. The possible reasons for this include the unfavorable physicochemical properties that some of these molecules have, as the case of CDST [81], which presents poor aqueous solubility while being a potent GOi (*K*_i_ = 15 nM). Besides, most of previous studies failed to reach assays at cellular or in vivo levels [85,104].

SRT constitutes a useful approach in inborn errors of metabolism which are characterized by substrate accumulation, as it is the case of PH1 [7,105]. For this reason, it was suggested that the inhibition of GO could lead to a reduction of the amount of glyoxylate to a point where residual AGT activity would be enough to prevent substrate accumulation [7]. Besides, the absence of GO in two brothers caused by a mutation in the gene *HAOX1* resulted in asymptomatic glycolic aciduria as sole manifestation, therefore highlighting the safety of a possible treatment based on GO inhibition [9]. Below, a brief outline of the development of GOi’s is given.

In order to determine the effect of the oral administration of GOi’s to animals, the GO inhibitory activity of pyrrole derivatives was explored by Merck Laboratories in 1983 [101]. Thus, a library of 4-substituted 3-hydroxy-1*H*-pyrrole-2,5-dione derivatives was prepared and tested for pig liver GO inhibition, showing a competitive inhibition pattern for this enzyme. Compound 4-(4′-bromo[1,1′-biphenyl]-4-yl)-3-hydroxy-1*H*-pyrrole-2,5-dione (Figure 5) was identified as the most potent hit within this set (IC_50_ = 87 nM for pig GO and IC_50_ =110 nM for hGO). Furthermore, its administration to rats fed with ethylene glycol resulted in a significant reduction in urinary oxalate output but only after a chronic administration period of 58 days during which there was no evidence of high toxicity [101].

With the aim of continuing the exploration of potential pharmacological treatments for PH1, molecular modeling and docking studies on sGO and hGO were performed with the flavonoids quercetin and kaempherol (Figure 5) extracted from *Tribulus terrestris* Linn (*Tt*) [104]. In vitro experiments with sGO revealed different inhibition patterns for these two flavonoids: quercetin behaved as a noncompetitive inhibitor of sGO (*K*_i_ = 0.56 µM and IC_50_ = 0.22 µM), and conversely, kaempherol was a competitive inhibitor of the enzyme (*K*_i_ = 0.37 µM and IC_50_ = 0.3 µM). Thus, these compounds were established as promising leads for future drug optimization.

The proof of concept for SRT in PH1 has been recently reported with the validation of GO as a safe and efficient target for SRT in PH1 mouse model using small molecules [7]. Within this study, the small GOi CCPST (Figure 4) was tested both in vitro and in vivo. In vitro, it behaved as a noncompetitive inhibitor, with a *K*_i_ value of 20.3 μM and an IC_50_ value of 43.5 μM against mouse GO (mGO). This compound was also able to significantly diminish the production of oxalate on *Agxt1*^−/−^ mouse hepatocytes (EC_50_ of 25.3 μM at 24 h). In the in vivo studies, the oral administration of CCPST at daily doses of 110 mg/kg body to *Agxt1*^−/−^ mice for 11 days resulted in a 30–50% reduction in urine oxalate without clinical signs of toxicity. However, in this study the need of more potent GOi’s was emphasized, since the high dose required for the treatment with CCPST could entail undesired side effects [7].

Other compounds such as colistimethate sodium have been identified as novel and more potent GOi’s, with IC_50_ value of 2.3 μM and showing a mixed linear inhibition pattern of hGO [47]. A cell-based assay with CHO-GO cells useful for high-throughput screening of compound libraries was also developed and optimized to test the activity of colistimethate sodium in cell culture, providing an IC_50_ value of 8.3 μM.

Very recently, we have identified salicylate derivatives (Figure 5) as mGOi’s and efficient agents that diminish oxalate production in *Agxt1*^−/−^ mouse hepatocytes [49]. The best mGOi’s, displaying IC_50_ values of 2−5 μM, carried π-deficient arenes in the hydrophobic tail and produced a moderate decrease of oxalate output in cell culture (20–30% oxalate decrease at 10 μM). Apart from π-deficient salicylate derivatives, it is worth to highlight that some furylsalicylates (Figure 5) were 7-fold more efficient than CCPST in oxalate output decrease in the cell-based assay. In this assay, the EC_50_ of approximately 3 μM that was achieved remained unaltered for 72 h and cytotoxic effects were not observed. However, their moderate mGO inhibitory activity (IC_50_ > 35 μM) suggested the presence of alternative biological target/s whereby these compounds might exert their oxalate decreasing effect and LDH was suggested as a plausible one. This is based in the fact that both enzymes can catalyze the transformation of the same substrate glyoxylate, and the salicylic derivatives were designed as substrate analogues. Moreover, in silico studies were used to analyze their binding mode and suggested that in flexible molecules with a V-shape conformation, the two aromatic moieties adopt a syn orientation that allows a more effective interaction. Altogether, this led to the establishment of preliminary structure-activity relationships for novel salicylates GOi’s with oxalate decreasing capacity. The salicylic acid polar head that bears free carboxy and hydroxy functionalities interacts at the binding region 1 and needs to be substituted on C5. On the other hand, electron withdrawing substituents are preferred in the aryl or heteroaryl hydrophobic tail that interacts with the second binding region, and a flexible linker can be introduced to space the polar head and the hydrophobic tail, therefore allowing a better accommodation of the molecule. This constitutes an unprecedented activity for salicylates, and their easy one-step synthesis along with their drug-like structure makes them promising candidates for drug development. Inherent to their salicylic structure, these compounds could exert a possible favorable anti-inflammatory activity or, on the other hand, a possible renal toxicity due to cyclooxygenase inhibition. These are issues under evaluation for these compounds [49].

Similarly, the company Orfan Biotech has explored triazole carboxylic acids and their esters as GOi’s [51], as well as pyrazoles, isoxazoles, isothiazoles, thiadiazols, and pyridazines [106]. Besides, their small GOi BBP-711 is currently in preclinical development [107].

### 2.2. Lactate Dehydrogenase Inhibitors

Lactate dehydrogenase (LDH; EC 1.1.1.27) is an enzyme of the family of the 2-hydroxyacid oxidoreductases thaµt can be found in almost all animal and plant tissues and in microorganisms. LDH plays a central role in several metabolic pathways and is mainly involved in the catalysis of the reversible conversion of pyruvate into lactate, coupled to the oxidation of cofactor NADH to NAD^+^ [108]. LDH in humans (hLDH) is a tetrameric molecule (140 kDa) that exists as different isoforms (isozymes) composed by the association of mainly two kinds of subunits (35 kDa): the M-type (also named LDH-A) and the H-type (LDH-B). Each subunit is encoded by different genes, which are *ldh-a* and *ldh-b* for M (muscle) and H (heart) subunits, respectively. The combination of A(=M) and B(=H) subunits gives up to five isozymes: the tetramers A_4_ (also named LDH-5), A_3_B_1_ (LDH-4), A_2_B_2_ (LDH-3), A_1_B_3_ (LDH-2), and B_4_ (LDH-1). LDH-1 (usually named hLDHB) and LDH-5 (hLDHA) are therefore homotetramers formed by four B-type or four A-type monomers, respectively, and are located mainly in heart and brain (hLDHB) and in skeletal muscle and liver (hLDHA) [109]. The subunits A and B are similar in size and share 75% of sequence identity, but the corresponding isozymes hLDHA and hLDHB show some kinetic and physiological differences [110]. For example, hLDHA has the highest activity in converting pyruvate into lactate under anaerobic conditions, whereas hLDHB is more efficient in the catalysis of the reverse reaction in well-oxygenated tissues [111]. Of all the isoforms, hLDHA has attracted great interest in recent years, as it was found to be overexpressed in a variety of highly glycolytic human cancers (see Section 2.2.1).

Although LDH was purified in crystalline form for the first time in 1940 from a heart muscle sample, it was not until 2001 that Brady and co-workers reported the first X-ray structure of hLDHA and hLDHB in a ternary complex with NADH and oxamate, a structural isostere of pyruvate and a well-known pyruvate competitive inhibitor (PDB codes 1I10, 1I0Z) [112]. Since then, several studies have followed on the X-ray crystal structure of hLDHA in apo and NADH binary complex forms (PDB codes 4L4R, 4L4S) [113], hLDHA in apo, ternary and inhibitor-bound forms (PDB codes 4QT0, 4QSM, 4OJN, 4OKN) [114] as well as on molecular modeling and molecular dynamics simulations [115,116,117], in order to understand the main binding modes of hLDHA. All this information has recently been reviewed [110,115,118] and it can be deduced that each monomer (subunit) of hLDHA has two domains, together with the N-terminal tail that provide a linkage with the C-terminal of the adjacent subunit, critical to allow the oligomeric assembly of the tetramer. The larger domain, formed by residues 20–162 and 248–266, is characterized by a ”Rossmann” fold [119], and is responsible for the interactions with the cofactor NADH (also referred as the “co-substrate binding” domain or site). The smaller domain, including residues 163–247 and 267–331, is responsible for key interactions with the substrate (also referred as the “pyruvate binding” domain or site) [109]. The two domains form a sort of bilobed structure where a central groove defines the enzyme active site. In addition, residues 95–110 form the so-called “active-site loop”, which is involved in the catalytic process and can adopt either an open or a closed conformation, thus regulating the accessibility of substrate and cofactor to the enzyme active site [115]. The cofactor binding site may in turn be divided in two sub-sites: (a) the nicotinamide binding pocket, hosting the nicotinamide moiety of NADH, which is located at the edge of the two domains and constitute the catalytic portion of the enzyme; (b) the adenine binding pocket, at the other side of the binding groove, which interact with the cofactor adenine moiety and the linked ribose ring. The substrate binding site is adjacent to the nicotinamide binding pocket where the catalyzed reaction occurs [115]. Mechanistically, hLDHA follows an ordered sequence of events in which NADH binds to the cofactor binding site, then pyruvate binds to the substrate binding site and, finally, the active-site loop closes to provide a largely desolvated ternary complex. It is then when a hydride ion is transferred from the nicotinamide ring of NADH to the carbonyl C-atom of the pyruvate [120].

The observed differences in catalytic activity between hLDHA and hLDHB mentioned above are thought to be, from a mechanistically viewpoint, the result of altered surface electrostatic interactions between binding sites and ligands. In fact, the p*Ka* of the active residue His193 increases from 7.3 in hLDHA (likely protonated at physiological pH) to 8.3 in hLDHB (non-protonated), representing a potentially exploitable distinction for rational design of hLDHA-specific inhibitors [112].

In addition to the well-studied reversible conversion of pyruvate into lactate by hLDHA, this isozyme is also involved in the conversion of glyoxylate into oxalate, the last step of oxalate metabolism in liver [3,121]. The mechanistic details of that conversion by the enzyme have not been studied yet but there are many evidence that support LDH as the key enzyme responsible for converting glyoxylate to oxalate, being recently reported the first in vivo proof in mouse models [55,56].

A liver organ selective inhibition of hLDHA is considered necessary to validate hLHDA as a safe therapeutic strategy in PH patients, thus avoiding likely non desired secondary effects [122]. Nowadays, there is new evidence that reinforces the idea that the use of small molecules as hLDHA inhibitors (hLDHAi’s) is a safe emergent therapy to different diseases (Section 2.2.1 and Section 2.2.2).

#### 2.2.1. Therapeutic Applications of Lactate Dehydrogenase Inhibitors

hLDH has been identified as a key enzyme in molecular mechanisms of different kind of disorders, such as cancer [123], vascular diseases [124], epilepsy [125], tuberculosis [126], pulmonary fibrosis [127,128], arthritis and other inflammatory diseases [129] and, more recently, in primary hyperoxalurias (PHs) [55,56].

Due to the known role of hLDHA in the “Warburg effect” in malignant cells [130], the inhibition of this enzyme has been considered as a strategy for diminishing the energy supply in cancer cells, reducing in that sense, the invasive potential of different kinds of cancer, such as breast carcinoma [131,132,133,134,135], pancreatic cancer [136,137,138,139,140,141,142,143,144,145], brain tumor [146], hepatocellular carcinoma [131,134,147], osteosarcoma [148,149,150,151], lung carcinoma [134,152,153,154], ovarian cancer [138], bladder cancer [155], colorectal cancer [134,138,155], mesothelioma [138], melanoma [134], leukemia [134], lymphoma [136], gastric cancer [156], Ewing’s sarcoma [145], leiomyomatosis, and renal cancer [157], achieving the in vitro*,* and in some cases in vivo*,* reduction of cancer cells.

This target and its applicability for cancer therapy has been extensively reviewed during the last few years, and a huge structural variability of hLDHAi’s with potential anticancer activity has been reported [108,109,110,111,118,123,146,158,159].

**Figure 6:**
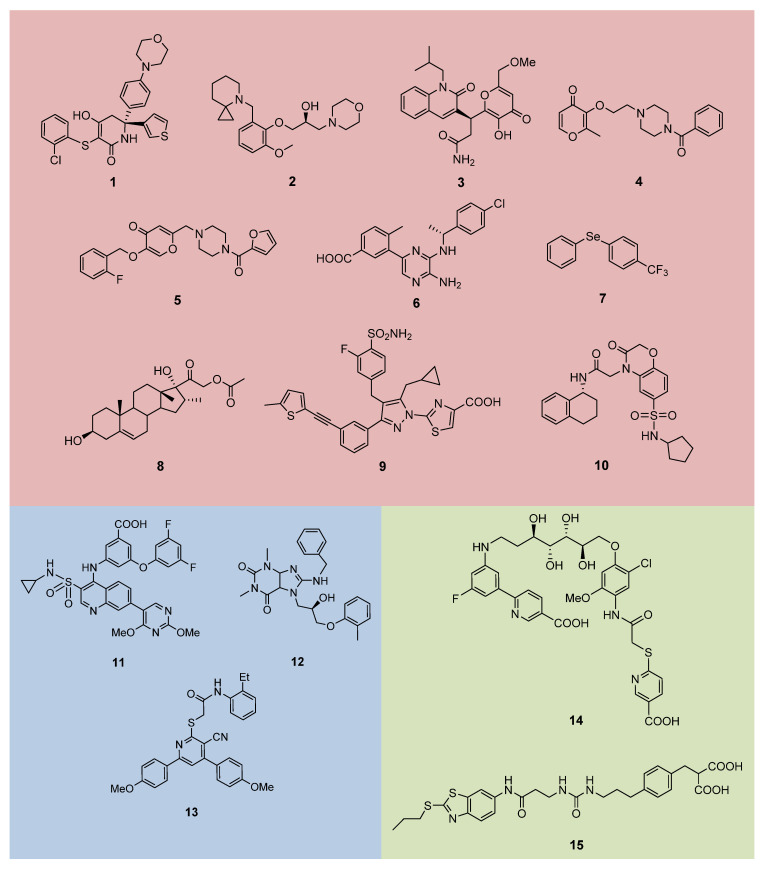
Structure of the most relevant hLDHA inhibitors according to its binding site: substrate (pyruvate) binding site (pink), cofactor (NADH) binding site (blue), substrate and cofactor binding sites (extended, green).

Table 1 and Figure 6 show the most relevant and active hLDHAi’s reported to date, designed and synthetized for each scaffold, and classified by its different binding mode to the enzyme, according to crystallographic data (PDB code) or molecular docking studies. As stated above, the active site of hLDHA comprises both a substrate binding site, which usually hosts small polar structures, and a cofactor binding site for NADH, which is more extended than that of the substrate and is composed of lipophilic as well as polar portions. Thus, compounds **1**−**10** are reported to bind the substrate-binding site, compounds **11**−**13** bind the NADH-binding site, and compounds **14** and **15** bind both sites at the same time. When crystallographic structures of hLDH in inhibitor-bound forms were not available, the structural information on the binding sites was used as templates for theoretical calculations and denoted in Table 1 as “docking” [117]. Moreover, other interesting data, such as their IC_50_ values against both hLDHA and hLDHB isozymes, EC_50_ values in cancer cell line assays and limitations or optimizations of these molecules for further in vivo experiments, denoted as “viability”, are also included in Table 1.

One of the main limitations observed in some of these inhibitors is their low selectivity to inhibit the hLDHA isoform. For instance, compounds **1** and **6** (Table 1; Figure 6) inhibit, with almost the same affinity, both isozymes hLDHA and hLDHB, which could cause secondary effects like cardiotoxicity since hLDHB is mostly found at heart muscle. This important secondary effect has hampered the continued development of these inhibitors [110,118]. Only compound **11** shows an adequate selectivity for both enzymes, being hLDHA preferably inhibited [131]. The inhibition selectivity for the rest of inhibitors included in Table 1 is not reported yet and should be evaluated before further development.

Another important limitation of these inhibitors is related to their pharmacokinetic (PK) properties. The small size and polar nature of the substrate site, and the long distance between the adenine and nicotinamide sub-pockets influence the structure of inhibitors synthetized, which are usually too big and highly polar and therefore make the process of crossing the cell membrane more difficult, diminishing their applicability as anticancer drugs [110]. In particular, the PK of very few hLDHAi’s have been studied up to now (compounds **1, 9** and **11**; Figure 6) and just compound **9** shows optimal PK profile with high cellular potency, in vitro ADME (absorption, distribution, metabolism and excretion), and in vivo PK properties [145].

Natural and synthetic compounds with a catechol moiety (e.g., gossypol, galloflavin, FX11) have been evaluated as hLDHAi’s and, despite their promising preliminary results, only some derivatives, such as **2**, could be suitable for further development [151]. Compound **2** and other inhibitors with 4*H*-pyran-4-one (**3–5**), selenobenzene (7), steroid (**8**), benzoxazine-6-sulfonamide (**10**), purine (**12**), and cyanopyridin-2-thioacetamide (**13**) scaffolds reduce cell viability in different cancer cells with a EC_50_ range between 1µM to 84 µM, but no selectivity and no pharmacokinetics behavior are reported for them (Table 1, Figure 6).

In spite of the great effort made in the design and synthesis of these new hLDHAi’s and the existence of various promising preclinical candidates, none of them have shown any real clinical benefit, although the recently reported compound **9** [145] could be the first one if its selectivity towards hLDHA over hLDHB proves adequate. In fact, there have been no clinical phase initiated based on these lead inhibitors, although some pharmaceutical industries, such as GlaxoSmithKline [131], AstraZeneca [163] and Genentech [142,160,164], have also been involved in the development of some of this kind of inhibitors.

It is clear that hLDHA has emerged as a very interesting target for anticancer therapy with substantial therapeutic potential and hence important efforts have been performed for developing potent hLDHA inhibitors. However, it is necessary to go one step further in order to improve their in vivo applicability by increasing the selectivity of inhibitors (hLDHA vs hLDHB), providing cell membrane permeability and reducing off-target interactions. Moreover, and due to the micromolar concentration of the LDH enzyme in cancer cells, an effective inhibitor will likely need to bind with exceptionally high affinity and selectivity and also achieve high intracellular concentrations to enable a therapeutic level of target engagement. To the best of our knowledge no inhibitors of hLDH matching these criteria have been reported yet.

#### 2.2.2. Lactate Dehydrogenase Inhibitors in the Treatment of PHs. Challenges: Isozyme Selectivity and Liver Selective Distribution

The participation of the liver cytosolic enzyme hLDHA in the last step of endogenous oxalate production, points at this one as a target in the development of molecular therapies for the treatment of the three type of PHs [3,55,121]. Lai et al. have demonstrated that inhibition of hLDHA with small interfering RNAs (siRNAs) leads to a profound effect on urinary oxalate excretion in mouse models of primary hyperoxaluria, PH1 and PH2 (*Agxt1*^−/−^ and *Grhpr*^−/−^, respectively) [55]. Therefore, subcutaneous injections in mice of *N*-acetyl-galactosamine (GalNAc) conjugated with siRNAs allowed a liver-specific knockdown of *Ldh-a* and no significant knockdown of *Ldh-a* in muscle, skin, or uterine tissue [55].

At this point, the search for small molecules with hLDHA inhibitory activity opens the possibility of a new advantageous treatment for PH, as a result of their stability and easy incorporation into pharmaceutical formulation suitable for oral administration. Racemic stiripentol, *rac*-(*E*)-1-(benzo[*d*][1,3]dioxol-5-yl)-4,4-dimethylpent-1-en-3-ol (*rac*-**1**), traded as Diacomit^®^ (Biocodex, Beauvais, France), is a third-generation antiepileptic drug approved by EMA in 2007 and the FDA in 2018 for the treatment of Dravet syndrome, and is being currently evaluated in other forms of epilepsy [165,166]. Several possible mechanisms of action have recently been recognized, including hLDHA inhibition [125]. Thereby, stiripentol was hypothesized to reduce hepatic oxalate production and, this way, hLDHA has been evaluated as therapeutic target for enzyme inhibitors against PH [14,54,57,167,168]. In a first study, a patient affected by severe PH1 was treated with this compound for several weeks showing a decrease of urine oxalate excretion without side effects [57]. However, the effectiveness of this drug seems to depend on the state of the renal function of the patient [167,168]. Forthcoming phase 2 clinical trial (NCT03819647) could raise further information on safety and efficacy of stiripentol monotherapy for the treatment of PHs [14,168,169]. These results obtained with racemic stiripentol make it deserving of additional studies in racemic resolution and evaluation of both enantiomers. To reach this goal, two strategies have been developed: (a) separation of stiripentol enantiomers on chiral stationary phase in high performance liquid chromatography (HPLC) [170]; (b) enzyme catalyzed kinetic resolution [171]. Moreover, our research group has carried out a third strategy based on the synthesis of the racemic (*E*)-1-(benzo[*d*][1,3]dioxol-5-yl)-4,4-dimethylpent-1-en-3-ol from two commercial compounds (3,4-methylenedioxybenzaldehyde and 3,3-dimethylbutan-2-one) using the Claisen-Schmidt reaction. The mixture of the enantiomeric alcohols obtained after reduction has been separated by means of derivatization with (–)-(1*S*)-camphanic chloride. Evaluation of the two separated enantiomers on hyperoxaluric mouse (*Agxt1*^−/−^, *Grhpr*^−/−^ and *Hoga1*^−/−^) hepatocytes is currently being performed [unpublished work].

**Figure 7:**
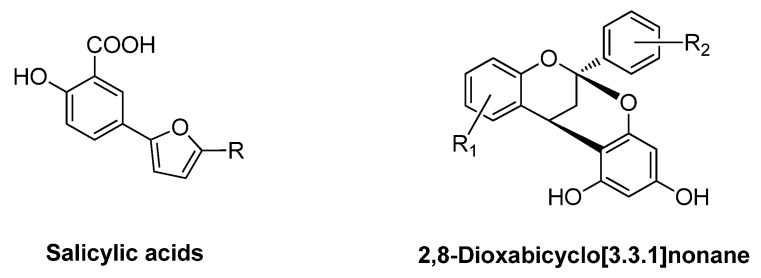
General structures of salicylic acid and 2,8-dioxabicyclo[3.3.1]nonane derivatives.

While small-molecule hLDHAi’s might provide an alternative to siRNA-based silencing, interest in developing more potent, selective and safer hLDHAi’s remains an attractive target. Recent advances in this sense, point at the use of active transport to introduce the inhibitors selectively inside the hepatocytes [172,173]. Nowadays, we are exploring the inhibitory activity of two families of compounds synthesized in our laboratories against both hLDHA and hLDHB isozymes. Thus, several salicylic acid and 2,8-dioxabicyclo[3.3.1]nonane derivatives (Figure 7) revealed satisfactory inhibition activities against hLDHA [unpublished work].

In order to enhance the applicability of hLDHAi’s as potential drugs for the treatment of PHs, it is very important to ensure its stability and suitable bioavailability to reach the hepatocyte cytosol with minimal secondary effects. Nowadays, the development of smart nanocarrier-based drug delivery systems is at the focus of the pharmaceutical technology, and different nanocarriers have been designed and evaluated [174]. Specific bioencapsulation strategies to target hepatocytes have been developed [175,176,177], and the use of polymeric micelles is one of the most attractive alternatives [178]. Their core-shell architecture wherein the hydrophobic core plays the role of a natural carrier for hydrophobic drugs and the hydrophilic shell allows the stabilization of the nanoparticle in aqueous solutions. Furthermore, their very low critical micelle concentration values favor its stability, even at low concentrations, which increase their circulation times in blood [178,179]. Toxicity and biocompatibility of polymeric micelles is a major feature and modified chitosan has been used widely due to its low toxicity, excellent biocompatibility and biodegradability [180,181]. The design of these smart nanocarriers requires an appropriate selection of the type of nanocarrier, targeting mechanisms to locate specific cells and stimulus techniques to release the drug inside. In this field, our research group is making some efforts in terms of design and synthesis of chitosan-modified polymeric micelles for targeting hepatocytes [182]. For that, two hepatocyte cell features have been taken into account: (a) the high expression level on hepatocytes of asialoglycoprotein receptors (ASGPR) vs minimal expression on extrahepatic cells [175,183]; (b) the different concentration of glutathione (GSH) in blood plasma (10µM) vs hepatic cells (10 mM) [177]. Thus, different types of ligands are used to decorate the chitosan micelle: deoxycholic acid as a hydrophobic moiety [183], polyethylene glycol as a protective agent, avoiding micelle removal by the reticuloendothelial system [174,181], galactose derivative as a specific liver-targeting ligand [177,184], and cystamine as redox-triggered burst drug release moiety [185]. GSH sulfhydryl group acts as endogenous stimulus, reducing the disulphide bonds of cystamine ligand and releasing the drug at the hepatocyte cytosol [174].

## 3. Issues in the Biological Testing of Enzyme Inhibitors against PH

### 3.1. Enzymatic Assays: Protocols and Challenges

Typically, both absorbance and fluorescence readouts are used in enzymatic and cell-based assays. When setting up an enzymatic assay, critical factors such as the selection of the appropriate fluorophore or the detection of colored compounds that could give undesired signals need to be considered to minimize the presence of interferences. This way, the assay will need to be adjusted to the library or specific assays will need to be implemented to detect possible interferences. Besides, the use of an alternative assay with a different detection method is desirable in order to verify the results [186]. In an enzymatic assay, key factors such as temperature, pH and ionic strength need to be taken into account, as well as the concentrations of all the assay components [187,188].

**Figure 8:**
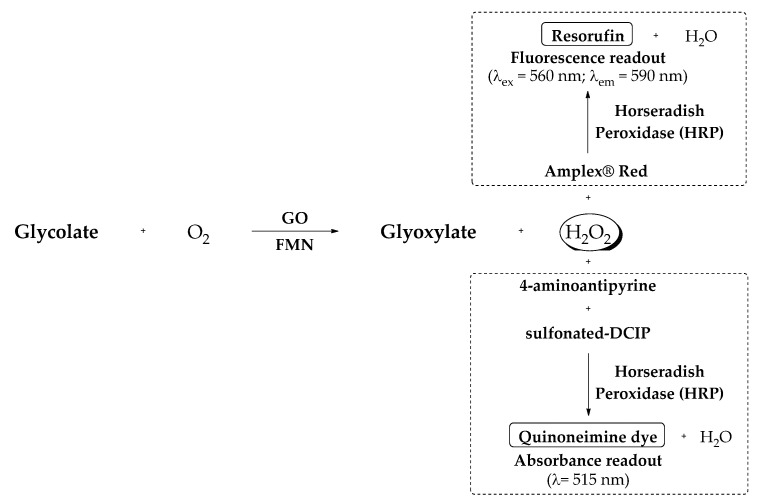
Colorimetric and fluorometric enzymatic assays for the evaluation of glycolate oxidase inhibitors (GOi’s).

Colorimetric and fluorometric end-point enzymatic assays have been developed to test the GO inhibitory activity. Both of them are based on the detection of H_2_O_2_ released after the GO reaction in the presence of glycolate as substrate. A colorimetric assay (Figure 8) was successfully implemented to test CCPST [7] and salicylate derivatives [49]. Their *m*GO inhibitory activity was evaluated using sulfonated dichloroindophenol (DCIP) as chromogen and 4-aminoantipyrine in a coupled horseradish peroxidase (HRP) reaction which yields a chromogen that is measured at 515 nm. Alternatively, following previous reported protocols to measure the activity of other oxidases [189,190], a new fluorometric assay (Figure 8) to measure the GO inhibitory activity was developed using fluorogenic Amplex^®^ Red reagent as reporter (Invitrogen, Eugene, OR, USA). Within this assay, H_2_O_2_ is quantified with coupled HRP reaction that yields fluorescent resorufin, whose fluorescence is measured with λ_ex_ 560 nm and λ_em_ 590 nm [47]. With this fluorometric assay, the presence of certain reagents, which may cause interferences, can be discarded. Besides, the substantial reduction of the amounts of reagents and enzyme used due to the higher sensitivity of fluorometric vs colorimetric assays, provides a reduction of the cost per assay.

Regarding LDH enzymatic assays, diverse commercial kits are available and different protocols to measure LDH activity in cell viability assays have been extensively documented in literature, considering the interest of LDH as an attractive target for cancer treatment. Described colorimetric protocols to assess cell viability determine LDH activity using a coupled enzymatic reaction, where LDH oxidizes lactate to pyruvate and this one reacts with iodonitrotetrazolium chloride (INT) to give formazan which absorbance is measured at 490 nm [191,192]. Alternative methods use the reverse reaction of reduction of pyruvate to lactate to measure NADH disappearance as it is oxidized into NAD^+^, and this process can be detected through the diminution of NADH absorbance at 340 nm [193,194,195]. However, compounds can easily interfere with the UV readout of NADH oxidation, and therefore the implementation of a kinetic fluorometric method is typically preferred [193]. Described kinetic fluorometric protocols measure NADH fluorescence in the presence of the substrate pyruvate at *λ_ex_* 340 nm and *λ_em_* 460 nm. Thus, the decrease of NADH fluorescence as it is oxidized to NAD^+^ can be easily monitored to obtain a negative slope which is smoothed when a LDH inhibitor is introduced in the assay [196,197]. Besides, this type of kinetic method allows the identification of compounds interfering with NADH fluorescence within the same assay, since the kinetic mode allows the correction of slight fluorescence interferences by subtraction of the baseline reading [196,197].

### 3.2. Cellular Models

Disease models are indispensable tools for understanding the molecular mechanisms that drive pathogenesis and enable the development of novel therapies.

#### 3.2.1. Mice Cellular Models of PH1, PH2 and PH3

Since the metabolic problem is centered in the liver but the main damaged organ is the kidney, cellular systems have major limitations to investigate the mechanisms of disease and explore potential therapeutic approaches based on our molecular understanding of the disease. Thus, genetically modified mouse models have been crucial for the recent advances in primary hyperoxaluria, providing the preclinical evidence needed to launch current clinical trials with patients, something that had been barely possible before the development of mouse models.

Five experimental molecular therapies have been proposed [121]: Substrate Reduction Therapy, Enzyme Replacement Therapy, Chemical Chaperone and Proteostasis Regulation Therapy, Gene Therapy and Cell Therapy. To investigate the physiopathology of PH and perform proof of concept studies that might lead to novel therapies, we cloned the mouse *Agxt* gene [198] and used standard gene targeting via homologous recombination in ES cells to generate an *Agxt*KO mouse model [20] that reproduced the main features of PH1, although with a milder phenotype than most human patients. Liver and kidney proteomic studies could be performed on mouse tissues to better understand the metabolic pathway involved in PH1 [199]. *Agxt*KO mice excrete high levels of oxalate in the urine, and their phenotype can be enhanced by challenging them with metabolic precursors, such as glycolate or hydroxyproline in the diet, at doses that induce nephrocalcinosis in the *Agxt*KO animals but not in the wild type controls.

We have also “humanized” the model, introducing human alleles as transgenes and resulting in animals with liver-specific expression of common human AGXT alleles, such as p.G170R, which results in mitochondrial mistargeting of the enzyme and p.I244T, the most prevalent mutation in the Canary Islands [200].

The *Agxt*KO mouse was used to demonstrate that the oxalate transepithelial flux in the gut could be used as a strategy to reduce the burden of oxalate in PH [65]. In fact, one of the first uses of our mouse model to test new therapies involved the testing of cross-linked recombinant enzymes to promote oxalate degradation in the gut [58]. Although limited benefits can be expected from this strategy in PH1 patients, which produce large amounts of oxalate in their livers, our proof-of-concept experiments were used for an innovative new drug (IND) application to the FDA and this approach is now being tested in patients with primary and secondary hyperoxaluria in ongoing clinical trials. Similarly, our PH1 mouse model has been used to test the potential of cell therapy in this orphan disease. Given the morbi-mortality and limitations of organs for liver transplant, the idea of using hepatocyte cell transplantation has been considered for inborn errors of metabolism for some time. We demonstrated that this strategy could have potential in PH1 provided that an efficient way to give the transplanted hepatocytes competitive growth advantage could be developed [201,202]. However, the cell-autonomous nature of oxalate overproduction in PH and the fact that diseased hepatocytes have no growth disadvantage, limits very substantially the application of this cell therapy [203].

Since all the forms of PH are due to liver enzymatic deficit and no irreversible neurological damage is part of the phenotype, gene replacement by gene therapy is a logical strategy that could yield curative results. Thus, we have used both adenovirus [20] and adeno-associated vectors (AAV5 and AAV8) [21] carrying the human *AGXT* sequence under the control of a liver-specific promoter to achieve curative results in *Agxt*KO mice. Long-term metabolic correction was achieved with AAVs and these preclinical studies were the foundation for an Orphan drug designation by EMA for a product currently in the pipeline of Uniqure (Amsterdam, Netherlands).

We have also used genetically modified mice to identify GO as a safe and efficient target for substrate reduction therapy [7]. Either deleting the GO gene or inhibiting its enzymatic product resulted in a substantial reversal of the hyperoxaluric phenotype of *Agxt*KO mice. We also found that siRNA inhibition of GO gene expression was very efficient reducing oxalate excretion in hyperoxaluric mice [8]. This therapeutic approach, explored in collaboration with industry, is currently being tested in a clinical trial with encouraging preliminary results. The availability of mouse models has allowed us to even explore very experimental therapies such as those using CRISPR/Cas9 tools in vivo [45], as a potential way of achieving PH1 cure by substrate reduction with a single administration of AAV-mediated GO-targeted guides.

As for PH2, we have also generated a *Grhpr*KO using a repository of gene-trapped ES cell lines [204]. Interestingly, the mouse model for PH3 that had been generated by KOMP consortium and we bred in our facilities, does not have the expected phenotype and it is being currently used to explore differences in mouse and human glyoxylate metabolism.

The *Grhpr*KO and *Agxt*KO mice have been also used to test the potential of LDHA siRNA to treat PH, regardless of the specific type of enzymatic deficit [55], providing the preclinical data necessary to embark in a clinical trial with PH patients that is ongoing.

#### 3.2.2. Human Cellular Models

The development of in vitro patient-specific disease models could be a very useful tool for the generation of more representative and relevant cellular models solving the species-specific differences between human and mice. In contrast to the wide range of cell culture models used in oxalosis research using renal epithelial cells, there is no cell culture model available to answer the questions related to primary human liver cell response to oxalate precursor or oxalate crystals exposure.

A novel strategy to generate patient-specific in vitro disease models is cell reprogramming, and in particular induced pluripotent stem cells (iPSC) generation, that opened a new avenue for disease modelling. The discovery that human fibroblasts could be reprogrammed directly to iPSC by forced expression of only four transcription factors [205] provided a new approach to disease modelling. iPSC can be derived from multiple somatic cell types obtained directly from individuals with the desired disease or from cell repositories. The ability of iPSC to model human diseases in vitro has revolutionized the ways in which monogenic, complex or epigenetic disorders are studied.

Reprogramming patient cells has several advantages over other strategies for the generation of disease-specific human models. The derivation of iPSC from multiple patients is usually straightforward, enabling the analysis of similar mutations in diverse genetic backgrounds. In addition, when modelling genetically complex disorders involving multiple unknown loci, patient-derived iPSCs are more beneficial than those based on genome editing of normal iPSC. Finally, patient-specific iPSCs may be helpful in making therapeutic decisions in the context of personalized medicine. Thus, many different diseases, including metabolic conditions, have been modeled generating iPSC [206,207]. In particular, iPSC from PH1 patients with the most common mutations (pG170R and pI244T) have been generated [16,208,209], demonstrating that they are a reliable tool for in vitro disease modeling and offering an advantageous system for the development of novel therapeutics and their use in drug screening.

One of the main motivations for generating models for human diseases is to develop therapies enabling the diseases to be treated, alleviated or cured. Animal models are frequently used for drug screening; however, many disorders lack a suitable animal model. Moreover, the use of animal models for high-throughput screening (HTS) of small-molecule libraries is usually not feasible. Thus, the use of iPSC disease models has become increasingly favored for purposes of drug discovery using HTS approaches. To achieve reliable efficiency, HTS usually require differentiation towards a specific cell type in culture with a phenotype that can be automatically measured and quantified. Based on appropriate protocols, iPSC can be differentiated to hepatocyte-like cells (HLCs) showing the expression of hepato-specific markers and with reliable hepatic functions [210,211]. PH1-iPSC-derived HLCs not only show hepatic functions, but also recapitulate some of the phenotypes associated with PH1.

Some studies have reported small-molecule screenings for PH [212]. Belostotsky et al. performed a drug screening using a quantitative Glow-AGT assay based on the self-assembly split-GFP approach to identify drugs that can correct mislocalization of the mutant G170R-AGT protein, the most frequent mutation in PH that results in aberrant mitochondrial localization of the active enzyme. They identified the translation elongation inhibitor emetine, demonstrating that a prolonged treatment corrected G170R-AGT mislocalization. These results, although performed in cellular models rather than using iPSC-derived patient-specific disease models, clearly indicate the potential that HTS could have in the identification of novel treatments for PH. Moreover, the possibility to use patient-specific iPSC-derived models would increase the relevance of the identified compounds.

In spite of the fact that no HTS have been reported using iPSC-derived HLCs for PH, small-molecule screenings with HLCs modeling hypercholesterolemia represent the proof of concept that these approaches are feasible [213]. In that study, HLCs generated from homozygous familial hypercholesterolemia iPSC were used to identify drugs that can potentially be repurposed to lower serum LDL-C. They found that cardiac glycosides reduce the production of apolipoprotein B from human hepatocytes in culture. These studies highlighted the effectiveness of using iPSC to screen for potential treatments for inborn errors of hepatic metabolism.

In conclusion, cell reprogramming and in particular iPSC-derived patient-specific disease models represent a reliable tool for the discovery of novel and innovative treatments for many different diseases, including PH. Moreover, the fact that PH1-iPSC are already available, together with the well-defined differentiation protocols to HLCs, open a window of brand new opportunities that need to be explored.

## 4. Conclusions and Outlook

As it has been mentioned above, lumasiran is a small interfering ribonucleic acid (siRNA) approved in November 2020 [214] for the treatment of PH1. It binds to the GO mRNA thus inducing its degradation and preventing the synthesis of this protein. This way it reduces the conversion of glycolate to glyoxalate as a SRT. In addition, it has been published that siRNA of LDHA also reduces the production of calcium oxalate in hepatic cells by reducing the production of LDHA into the liver [55,56].

One of the concerns of using a biological drug, particularly siRNA, in a life-long repeated administration is the lack of clinical experience with this kind of treatments when compared with the use of classical small-molecule drugs for other diseases. Given the success of the lumarisan strategy in the PH1 treatment, it emphasizes the idea that a small molecule GO inhibitor development could also be effective [7,49]. The latter could also benefit from economic reasons and an oral administration, among others. The inhibition of GO is also supported by the identification of a healthy adult with GO knockout [10,11].

LDH has been reported as the main enzyme that catalyzes the conversion of glyoxylate to oxalate. Moreover, plasma glycolate was not elevated after the administration of siRNA for LDHA as it was observed after the administration of siRNA for GO [55]. Moreover, no liver toxicity was observed after the siRNA for LDHA treatment. Altogether this supports the idea of the development of a small molecule inhibitor of LDHA. However, this inhibitor must be isozyme selective and liver specific to avoid the problems already described in patients with hereditary LDHA deficiency [215,216], among others. This has been addressed with the development of the LDHA inhibitor CHK-336 [173]. It is an orally active tight binding inhibitor with an IC_50_ below 1 nM that uses the organic anion transporting polypeptide (OATP) transporter [172] and has low passive permeability. CHK-336 could be used to treat all three forms of PH and phase I clinical trial is planned to begin in 2021.

Another focus to debate will be the ideal type of enzyme inhibitor (GO and/or LDHA) including covalent vs noncovalent inhibitors [217,218,219]. The aim of a selective covalent binding to the enzyme is the target silencing. The administration of lumarisan (once quarterly or monthly after the first three months) invites to think on the development of covalent based inhibitors of GO. On the other hand, the initial results reported on CHK-336 with a very slow off-rate [173] do not exclude that a covalent inhibitor could benefit the patient. In either case, the structure of the covalent inhibitor may be modulated to optimize the residence time [220] using a reversible targeted covalent inhibitor [219]. Moreover, the structure can be modified in order to reduce the possibility of adverse reactions [218].

The clinical use of enzyme inhibitors in rare diseases has been demonstrated [221]. Miglustat and eliglustat [222,223] are inhibitors of the enzyme glucosylceramide synthase that are being used in SRT for type 1 Gaucher’s disease. Ecallantide [224] is a drug used in hereditary angioedema that inhibits the protease kallikrein. Nitisinone [225] is a competitive inhibitor of the enzyme 4-hydroxyphenylpyruvate dioxygenase approved for the treatment of hereditary tyrosinemia type 1. It is also currently being evaluated for the treatment of alkaptonuria [226].

The strategy of drug repurposing [227] has afforded a possible new indication for an already marketed anticonvulsant drug. Stiripentol highlights the suitability of a small molecule LDHA inhibitor for PH1 treatment [53,57], after it was noticed that patients receiving stiripentol had a lower urine oxalate excretion. Though a significant reduction of urine oxalate excretion was attained after one PH1 patient received stiripentol [57], this drug failed to lower the plasma oxalate concentration in a PH patient with advanced chronic kidney disease [167]. These two independent research groups used different surrogate end points that may explain the differences in the outcome of the investigation [228]. The ongoing phase 2 clinical trial NCT03819647 [169] evaluates the efficacy of stiripentol for the treatment of PH [168,229]. The enrollment of more patients may clarify the clinical limits for a treatment using this enzyme inhibitor. A patent application has been filled for analogs of stiripentol although complete results have not been published [230].

The fact that the product of GO is the substrate of LDHA prompts the idea of using both strategies looking for synergy. This may be traditionally envisioned using only small molecule drugs, but a treatment based on siRNA and a small molecule drug could also act synergistically [231]. Clinically, this combination would imply a dose reduction of each drug thus reducing adverse drug reactions (including long term developed drug reactions) and may allow solving the variability in disease characteristics between patients [232].

## Figures and Tables

**Table 1:** Activity and viability of the most active hLDHA inhibitors.

Bind.Site ^1^	Scaffold	Hit Number (PDB code)	IC_50_ (*h*LDHA); (*h*LDHB)	Cancer Cells (EC_50_)	Viability ^2^	Ref.
Substrate (pyruvate)	Hydroxylactam	**1** (4ZVV) ^3^	3 nM; 5 nM	Pancreatic (670 nM)	a, b	[142,143]
	Catechol	**2** (docking)	390 nM; n.d.	Osteosarcoma (3.2 µM)	a, b	[151]
	4*H*-Pyran-4-one	**3**–**5** (docking)	90–330 nM; n.d.	Panel of cancer cells (2.1–13.2 µM)	a, b	[144,150,154]
	Pyrazine	**6** (4M49)	0.5 µM; 2 µM	n.d.	a, b	[154,160]
	Selenobenzene	**7** (docking)	145 nM; n.d.	Panel of cancer cells (45–84 µM)	a, b	[134]
	Steroid	**8** (docking)	360 nM; n.d.	Lung (3–6 µM)	a, b	[152]
	Pyrazole	**9** (docking)	40 nM; n.d.	Pancreatic (119 nM) Ewing’s sarcoma (105 nM)	a, c	[145]
	Benzoxazine-6- sulfonamide	**10** (docking)	1.5 µM; n.d.	Pancreatic (3.2 µM)	a, b	[141]
Cofactor (NADH)	Quinoline-3- sulfonamide	**11** (docking) ^4^	3 nM; 43 nM	Hepatic (2.9 µM)	b	[131]
	Purine	**12** (docking)	250 nM; n.d.	Breast (1.5 µM)	a, b	[161]
	Cyanopyridin-2- thioacetamide	**13** (docking)	1 µM; n.d.	Osteosarcoma (1 µM)	a, b	[148]
Extended	Bifunctional ^5^	**14** (4I9H)	120 nM; n.d.	n.d.	a, b	[162]
	Bifunctional ^5^	**15** (4AJN)	270 nM; n.d.	n.d.	a, b	[163]

^1^ Binding site: substrate (pyruvate) binding site (pink), cofactor (NADH) binding site (blue), substrate and cofactor binding sites (extended, green). ^2^ (a) Low selectivity or not reported; (b) non-optimal pharmacokinetic profile or not reported; (c) further optimizations are ongoing for in vivo studies. n.d.: Not determined. ^3^ Compound **1** (GNE-140, Genentech, Inc.). ^4^ Compound **11** (GSK 2837808A, GlaxoSmithKline, Inc.). ^5^ Bifunctional compounds are molecules with two portions (one simulating the substrate and the other mimicking the cofactor) that are conjugated by a linker to cover the whole binding pocket of the enzyme.

## Data Availability

No new data were created or analyzed in this study. Data sharing is not applicable to this article.
